# Cerebrospinal fluid and plasma cytokines after subarachnoid haemorrhage: CSF interleukin-6 may be an early marker of infection

**DOI:** 10.1186/1742-2094-9-255

**Published:** 2012-11-23

**Authors:** Stephen J Hopkins, Catherine J McMahon, Navneet Singh, James Galea, Margaret Hoadley, Sylvia Scarth, Hiren Patel, Andy Vail, Sharon Hulme, Nancy J Rothwell, Andrew T King, Pippa J Tyrrell

**Affiliations:** 1 The University of Manchester Stroke & Vascular Centre, Manchester Academic Health Science Centre, Salford Royal Hospitals Foundation Trust, Eccles Old Road, Stott Lane, Salford, M6 8HD, UK; 2 Faculty of Life Sciences, University of Manchester, AV Hill Building, University of Manchester, Oxford Road, Manchester, M13 9PT, UK; 3 Department of Neurosurgery, Leeds General Infirmary, Great George Street, Leeds, LS1 3EX, UK

**Keywords:** Cerebrospinal fluid, Cytokines, Infection, Interleukin-6, Markers, Plasma, Subarachnoid haemorrhage, Ventriculostomy

## Abstract

**Background:**

Cytokines and cytokine receptor concentrations increase in plasma and cerebrospinal fluid (CSF) of patients following subarachnoid haemorrhage (SAH). The relationship between plasma and CSF cytokines, and factors affecting this, are not clear.

**Methods:**

To help define the relationship, paired plasma and cerebrospinal fluid (CSF) samples were collected from patients subject to ventriculostomy. Concentrations of key inflammatory cytokines, interleukin (IL)-1ß, IL-1 receptor antagonist (IL-1Ra), IL-1 receptor 2, IL-6, IL-8, IL-10, tumour necrosis factor (TNF)-α, and TNF receptors (TNF-R) 1 and 2 were determined by immunoassay of CSF and plasma from 21 patients, where samples were available at three or more time points.

**Results:**

Plasma concentrations of IL-1ß, IL-1Ra, IL-10, TNF-α and TNF-R1 were similar to those in CSF. Plasma TNF-R2 and IL-1R2 concentrations were higher than in CSF. Concentrations of IL-8 and IL-6 in CSF were approximately10 to 1,000-fold higher than in plasma. There was a weak correlation between CSF and plasma IL-8 concentrations (r = 0.26), but no correlation for IL-6. Differences between the central and peripheral pattern of IL-6 were associated with episodes of ventriculostomy-related infection (VRI). A VRI was associated with CSF IL-6 >10,000 pg/mL (*P* = 0.0002), although peripheral infection was not significantly associated with plasma IL-6.

**Conclusions:**

These data suggest that plasma cytokine concentrations cannot be used to identify relative changes in the CSF, but that measurement of CSF IL-6 could provide a useful marker of VRI.

## Background

Experimental and clinical studies of traumatic brain injury and stroke have identified inflammation as an important element of the pathological response [1,2]. In clinical studies, the relationship between clinical status and inflammation has commonly been established by measuring inflammatory markers in plasma or cerebrospinal fluid (CSF) [3-6]. Collection of CSF is rarely indicated clinically in conditions such as ischaemic stroke, but is possible in patients with traumatic injury or subarachnoid haemorrhage (SAH), where CSF from the lateral ventricles can be sampled over time in those who, for clinical reasons, have had an external ventricular drain (EVD) inserted. In a number of clinical studies of both stroke and SAH, inflammation within the CNS has been imputed by detection of increased inflammatory markers in plasma. Support for the validity of this derives from clinical studies that have described an association between plasma inflammatory markers and outcome [7-9]. The relationship between inflammatory markers in CSF and plasma is, however, uncertain. Do the increased plasma markers originate primarily in the brain or are they generated peripherally? Could it be that plasma markers identify systemic inflammation, or inflammation at the vascular interface, as an important determinant of outcome, relative to inflammation apparent from CSF data? Addressing these questions has important mechanistic implications in terms of understanding the relative importance of central and peripheral inflammation in these conditions.

The primary objective here was therefore to define the relationship between central and peripheral markers of inflammation, following SAH. To achieve this we undertook a prospective study to compare peripheral (plasma) and central (CSF) inflammatory markers in patients with SAH. This was conducted as part of a larger study of delayed cerebral ischaemia in patients with SAH [10] and took advantage of the availability of CSF from patients who had EVDs placed as part of their clinical care. Patients with EVDs are often critically ill and at high risk of infections. Since infection is also a potent inflammatory stimulus, a secondary, retrospective analysis was undertaken to examine the extent to which peripheral infection or ventriculostomy-related infection (VRI) was associated with observed changes in peripheral or central IL-6.

## Methods

### Patient inclusion criteria and data collection

Patients with SAH were eligible for this study if they presented within 7 days of symptom onset to the Greater Manchester Neuroscience Centre at Salford Royal NHS Foundation Trust and required placement of an external ventricular drain (EVD). All but three patients were recruited consecutively from a cohort that is reported in detail elsewhere [10]. After this, three additional patients were recruited consecutively, under the same conditions, since we had a target of at least 20 patients. Four patients only were excluded, on the basis that less than three paired blood and CSF samples were collected. Prior ethical approval was obtained from the Bolton research Ethics Committee, via the National Research Ethics Service, and the University of Manchester Research Ethics Committee. Informed consent was obtained from each patient or their representative.

Adult patients (>18 years) with a confirmed diagnosis of SAH (CT imaging or positive xanthochromia) were identified between January 2004 and June 2006. Eligibility was restricted to those with an underlying aneurysm who underwent formal angiography.

A detailed record of clinical presentation was made. This included presenting features, modified Rankin score, World Federation of Neurosurgeons Scale (WFNS), Fisher grade, Glasgow coma score aneurysm size and location, detailed demographics, infection and medication screen, atherosclerotic risk factors and physiological parameters. Daily clinical assessments included changes in medication, GCS, temperature, pulse rate, white cell count (WCC) and other evidence of infection.

A 2 mL CSF sample was collected at time of EVD insertion, where possible, and a concurrent 10 mL sample of blood was collected into 100 units of pyrogen-free heparin. Samples were cooled to 4°C and centrifuged for 15 min at 2,000 g. Plasma and CSF were decanted and stored at −70°C, prior to analysis of inflammatory markers. Clinical assessments were continued on a daily basis and further paired CSF and plasma samples were collected between 07:00 and 11:00, until 7 days after EVD insertion, where possible.

### Infection status

Infection status was determined from retrospective analysis of patient records, including the infection screening and antibiotic records on the study clinical report form. Definition of infection was divided into: (1) Definite: for peripheral infection this required positive culture, plus clinical picture of infection, raised WCC or commencement of antibiotics, while for VRI a positive culture with a clinical picture of infection or commencement of intrathecal antibiotics was necessary; (2) Probable: for peripheral infection this was positive culture in the absence of clinical picture or raised WCC/ CRP, but where antibiotics were not started; or negative culture in the presence of a clinical picture of infection or raised WCC/CRP, where antibiotics were not started. For VRI this was raised CSF WCC: red cell count ratio (>1:700), in the absence of positive culture or starting intrathecal antibiotics, but with a clinical picture of infection; (3) Unlikely: very limited documentation suggesting infection; and (4) No infection: no documentation suggesting infection, or documentation that excluded infection. Only those infections that were present at study entry, diagnosed during collection of samples, or diagnosed up to 2 days post sampling were counted as relevant.

### Cytokine assays

Measurement of interleukin-1ß (IL-1ß), sIL-1R2, TNF-α, sTNFR1, sTNFR2 and IL-10 was performed as described previously [9]. IL-1Ra was also measured as described previously [11] The IL-6 assay was undertaken essentially as in Emsley *et al.*[12], but instead using PeliPair anti-IL-6 antibodies (M9316, Sanquin, Amsterdam, the Netherlands) and development with Zymed steptavidin-horseradish peroxidise conjugate (ZyMax grade, Zymed Laboratories, San Francisco, CA, USA). IL-8 was measured similarly, but using BioSource Cytoset^TM^ antibodies and streptavidin (Cat. No. CHC1304; BioSource, Nivelles, Belgium). All cytokines were standardised in the laboratory against the latest respective national or international standards (National Institute for Biological Standards and Control, South Mimms, UK).

### Statistical analysis

Following log transformation, Pearson’s correlation was used to calculate the relationship between peripheral and central IL-6 or IL-8. To identify the relationship between IL-6 concentration and infection, comparison was made between either cases defined as having definite evidence for infection and those with no evidence, or between those with definite or probable evidence for infection and those with no evidence or unlikely infection. Logistic regression was undertaken, both for trend with increasing peak IL-6 values and in respect of a threshold IL-6 concentration, chosen from initial inspection of the data, to determine the odds ratio for association between IL-6 and infection. Since absence of high CSF IL-6 perfectly predicted absence of infection, logistic regression could not be applied as intended and Fisher’s ‘exact’ test was substituted.

## Results

### Samples analysed

Twenty-five patients were recruited to the study. The median age was 53 years (range, 36 to 72 years) and 14 (56%) were women. The aneurysm was anterior circulation in 20 and posterior circulation in four, <10 mm in 20 and, 10 to 25 mm in four (missing data from one). Sixteen patients underwent coiling, three clipping, one clipping after failed coiling, and four were treated conservatively (missing data from one). The Fisher grade was 3 in 14 and 4 in 11. The WFNS grade was I in 6, II in 11, III in 2, IV in 1 and V in 5. The median number of days from ictus to first sample collection was 2 (range, 0 to 10 days). Matched plasma and CSF samples for at least three time points were available from 21 patients.

### Cytokines and receptors that were similar, or higher, in plasma compared to CSF

**Figure 1:**
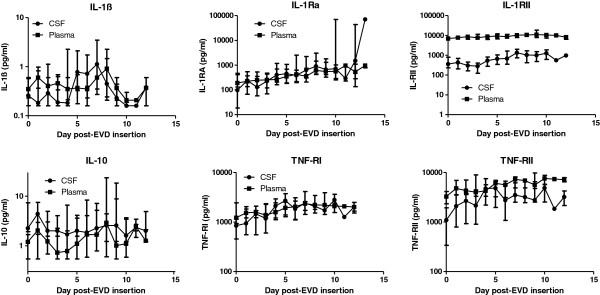
**The median (IQR) values for cytokines and cytokine receptors in paired plasma and CSF samples from 21 patients.** All time points were not available for all patients but are as identified in Figures 3 and 4 .

Concentrations of TNF-α were detectable in <30% of CSF samples and <10% of plasma samples (<0.6 pg/mL; not shown). Concentrations of IL-1ß in plasma were >2 pg/mL in only two samples from a single patient. Although 15 CSF samples had concentrations greater than this, and three samples contained between 10 and 40 pg/mL, the mean concentrations were similar to those of plasma (Figure 1). The IL-1 and TNF receptor concentrations, particularly in CSF, were remarkably similar between individuals, as indicated by the relatively small interquartile range. The pattern for IL-1Ra was similar to IL-1ß, although the concentrations were approximately two orders of magnitude higher. A similar pattern was seen with TNFR1 and IL-10, although IL-10 was consistently slightly higher in CSF. For TNFR2 the plasma concentrations were approximately three-fold higher than in CSF and for IL-1R2 the plasma concentrations were about an order of magnitude higher than in CSF.

### High CSF IL-6 and IL-8 concentrations compared to plasma

**Figure 2:**
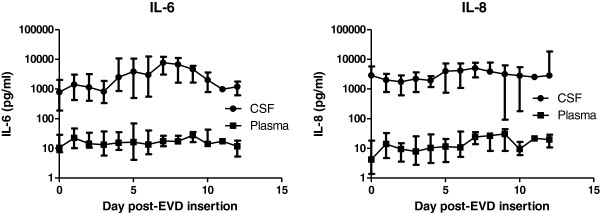
**The median (IQR) values for IL-6 and IL-8 in paired plasma and CSF samples from 21 patients.** All time points were not available for all patients but are as identified in Figures 3 and 4 .

**Figure 3:**
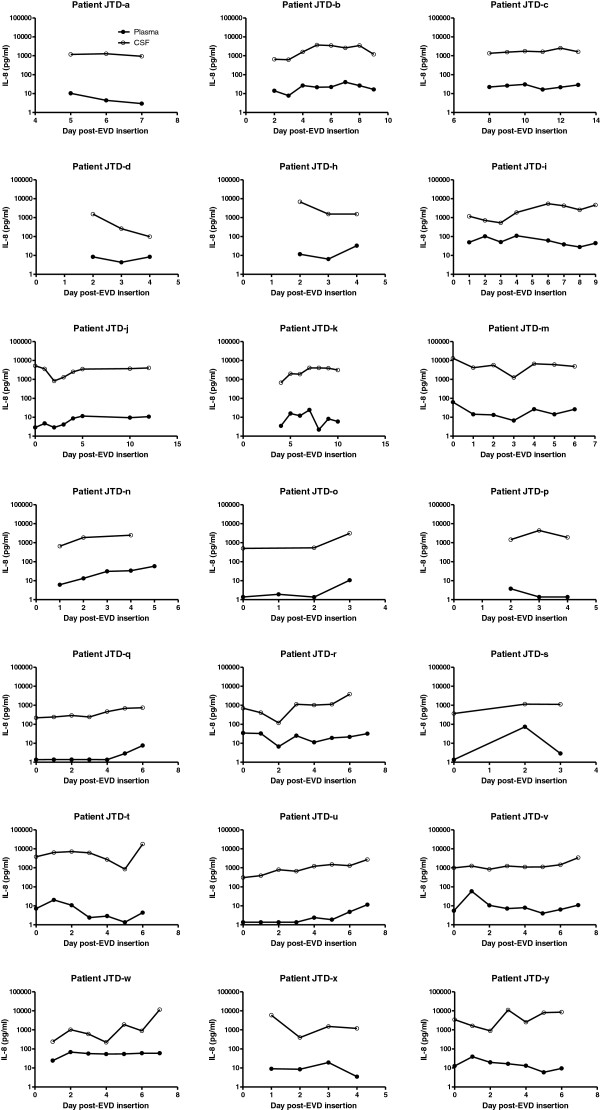
Paired plasma and CSF samples from 21 patients were analysed for IL-8 at the times indicated.

The IL-6 and IL-8 concentrations in CSF were between one and three orders of magnitude higher than in plasma (Figure 2). In most (97%) paired samples, IL-8 CSF samples remained an order of magnitude greater than plasma (Figure 3). There were very different ratios of plasma and CSF IL-8 between individuals. Correlations for individuals ranged from −0.51 to 0.99, with a mean of 0.26.

**Figure 4:**
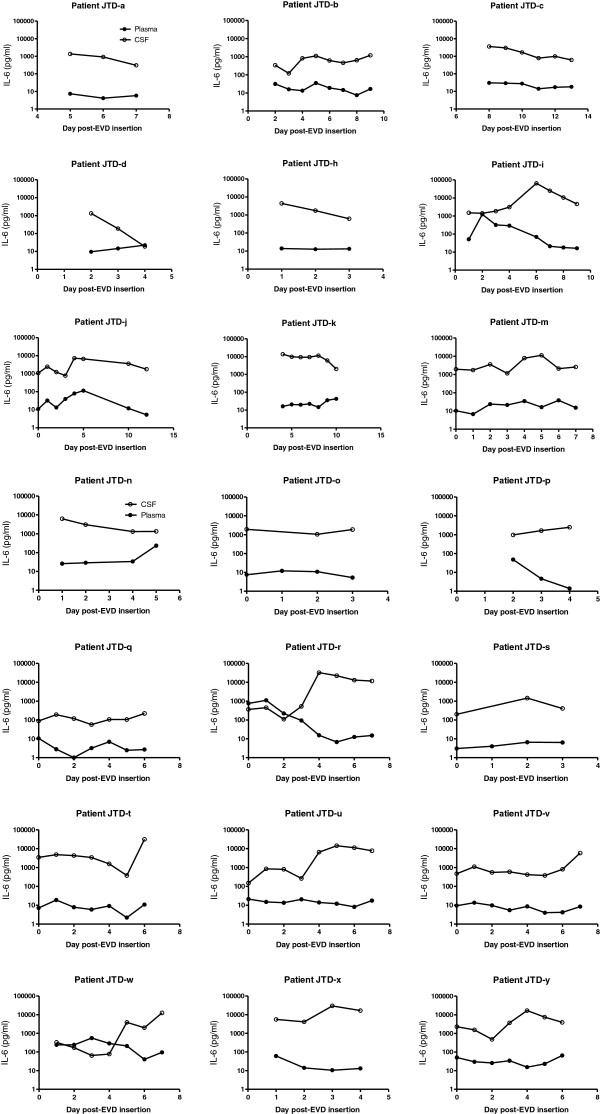
Paired plasma and CSF samples from 21 patients were analysed for IL-6 at the times indicated.

Although plasma and CSF patterns for IL-6 were remarkably similar to IL-8 in most cases, the order of magnitude difference in concentrations between CSF and plasma was maintained in slightly fewer (88%) of the paired samples (Figure 4) and plasma IL-6 concentrations less consistently tracked those in CSF. Correlations for individuals ranged from −0.96 to 0.90, with a mean of −0.18.

### Association between IL-6 and infection

When patients with definite VRI were compared to those with no evidence of VRI (Table 1), the test for increasing odds with increasing IL-6 was incalculable as prediction was perfect. Concentrations of IL-6 in CSF >10,000 pg/mL were significantly associated with VRI (Fisher’s: *P* <0.001). When cases of probable VRI were combined with definite VRI, and cases of unlikely infection were combined with those having no evidence of VRI, the test for trend was also significant (*P* = 0.04). The association with high IL-6 concentration remained strongly significant (Fisher’s: *P* <0.001). When peripheral infection (Table 1) was analysed in a similar way, with a cutoff for IL-6 at 30 pg/mL, the odds ratio was not significant for any of the analyses.

**Table 1:** Association between high CSF IL-6 and CSF infection

**CNS infection**	**CSF IL-6**	
	**High (>10,000 pg/mL)**	**Low (<10,000 pg/mL)**
Definite	i, k, t, u, x, y	
Probable	w	
No	m	a, b, c, d, h, j, n, o, p, q, s, v
Unlikely	r^a^	

^a^No CSF taken for culture. Patient letters correspond to those in Figures 3 and 4.

**Table 2:** Association between high plasma IL-6 and peripheral infection

**Peripheral infection**	**Plasma IL-6**	
	**High (>30 pg/mL)**	**Low (<30 pg/mL)**
Definite	b, c, i, j, m, n, r, w	s, t, u, v
Probable	k	
No	p, x	h, q
Unlikely	y	a,d,o^a^

^a^Patient letters correspond to those in Figures 3 and 4.

## Discussion

Clinical studies of the role of inflammation in brain tissue rely on determinations of biomarkers from clinically accessible fluid compartments. Plasma is clearly the most accessible and can be measured repeatedly to determine changes over time. We have shown that the concentrations of many of the commonly measured markers were similar in plasma and CSF compartments and in some instances plasma markers were higher. Direct comparisons with studies reported previously are problematical, since the assays differ and virtually none make reference to recognised standards. CSF IL-1ß has been reported as undetectable, or low and stable, in some studies of SAH [3,13], while another study reported it as increased, but in the low picogram range [4], and two others recorded tens to hundreds of picograms per millilitre [5,6]. TNF-α has similarly been found to be low or undetectable in some studies [4,13], but increased in others [6,14]. As in our study, IL-1Ra and TNFR1 concentrations have been reported as similar in CSF and plasma or serum, but with an increase after SAH in at least some patients [14,15].

In contrast, CSF concentrations of IL-6 and IL-8 were several orders of magnitude greater than those in plasma after SAH. Other studies have consistently found that CSF concentrations of IL-6 and IL-8 are increased one or two orders of magnitude above those in normal CSF (taken preoperatively for spinal anaesthesia, during elective aneurysm clipping or for diagnostic purposes that subsequently found no inflammatory cause) or plasma values [3,4,6,13,16,17]. There was a superficially steady relationship between plasma and CSF concentrations for individual patients, particularly for IL-8. However, the relationship between these compartments in different individuals varies considerably, as indicated by the poor correlation between compartment values for the cohort. This could reflect variability between individuals in the transport of cytokines from CSF to plasma, or simply be due to production occurring discretely in each compartment. The probability of the latter explanation being correct is suggested by the dissociation between plasma and CSF IL-6 following infection.

The absence of a gradient concentration between CSF and plasma for most markers, and the lack of a clear relationship between IL-6 or IL-8 in CSF and plasma, suggest that plasma concentrations of inflammatory markers cannot provide useful information about inflammation in the brain. Several neurosurgical studies have used microdialysis to address this issue, including a study in patients with SAH [18,19]. However, apart from the difficulty of placing the dialysis probes such that they provide a representative sample of events taking place in damaged tissue, collected data may reflect tissue damage caused by inserting the probes. It is notable that in a study where a significant change was seen over the collection period (for IL-6, IL-8, macrophage inflammatory protein 1ß and fibroblast growth factor 2) the highest values were observed in the first 6 h following probe insertion and these values fell over the next 24 h [19].

It may be that systemic inflammation and plasma markers are of greater significance than markers detected in the CNS, perhaps because this reflects neurovascular involvement. This is borne out to some extent by the reported associations between systemic inflammation or peripheral inflammatory markers and clinical outcome in situations following brain damage [7,20-23].

Our observations additionally raised questions as to what might be causing some of the changes in cytokine concentration within compartments and altering the relationship between central and peripheral concentrations, particularly for IL-6. After reviewing the clinical report forms and patient notes the most consistent other event occurring post-EVD insertion was infection. It is noteworthy that rather little mention is made of infection in study reports or commentaries on the association between cytokines or inflammation following SAH. Infection is a classic activator of inflammation and immunity, and bacterial products are widely used to activate inflammatory responses in experimental settings [24-27]. Ignoring the impact that infection may have on measures of inflammation seems surprising in any study of inflammatory markers, but perhaps particularly in conditions where infection is prevalent. Quite apart from its potential to induce production of cytokines, infection could modify cytokine production occurring as a result of the underlying pathology or might independently have an important influence on clinical outcome. Infection therefore has the potential to obscure relationships between a clinical condition and inflammation, or to suggest associations where both outcome and inflammatory markers are independently influenced by infection.

Depending upon the assessment measures used, infection could potentially alter clinical outcome in several ways. It may influence outcome by directly affecting body systems not threatened by the underlying clinical condition of interest, or modify metabolic functions, including vascular or renal function, such that the primary pathology progresses more rapidly. Alternatively, the infectious stimulus may exacerbate inflammatory responses associated with the condition being investigated. In this respect, several studies have shown that infectious stimuli increase the severity of CNS ischaemic pathology attributable to inflammation [28,29].

Since each of the measured markers has been associated with inflammation it might be expected that most would be similarly affected by infection. Considering the remarkably similar production pattern of IL-6 and IL-8 in these SAH patients, it is perhaps particularly surprising that IL-8 production was quite resistant to modification by infection. Induction of IL-6 seemed particularly sensitive to infection in the patients studied and these data suggest that monitoring IL-6 might be used as a marker of infection.

The association between IL-6 and peripheral infection has been well described ([25-27,30,31] and has been indicated as of value in diagnosis [32-34]. It is important to note that ours was a *post-hoc* analysis, driven by observed data, and therefore likely to overstate the association. Nevertheless, the strength of association is striking and merits further confirmatory studies. Elsewhere, CSF cytokines have been evaluated to diagnose infection in a variety of neurosurgical conditions and IL-1 and TNF were suggested to be of use, whereas IL-6 and IL-8 were not [35]. This contrasts with our study, where IL-1 or TNF were barely detectable and only IL-6 production was consistently modified by infection.

At least three other studies have considered the value of monitoring CSF IL-6 to aid the diagnosis of VRI. Of these, measurement of CSF in four pre-term infants with hydrocephalus suggested that it may be useful [36]. Analysis of IL-6 in 21 adult patients with EVDs concluded that IL-6 was not of value [37]. However, a subsequent study in 75 patients with EVDs concluded that IL-6 had significant value for predicting VRI 1 day before other diagnostic markers [38].

The apparent lack of consensus on how VRI is diagnosed highlights the need for an improved method for discrimination. Even positive culture is not definitive and may be identified as due to catheter colonisation or contamination in the majority of cases [39]. Similarly, lack of consensus on use of prophylactic antibiotic cover during use of intracranial monitors and EVDs [40], combined with current concern over inappropriate use of antibiotics, identifies the need for improved, early identification of infection. This study suggests that there would be value in clarifying whether rapid IL-6 measurement may provide a sufficiently sensitive and specific marker to identify VRI early and make a better informed decision on antibiotic use.

## Conclusions

The minor differences between some CSF and plasma cytokines and receptors we identified, combined with the relatively short half-lives of plasma cytokines, do not support the concept that plasma markers are primarily derived from the brain. Further, in the case of cytokines such as IL-6 and IL-8, where there is a significant difference in CSF and plasma concentrations, and these differences are relatively stable within individuals, the variability of the difference between individuals means that it is not possible to deduce differences in CSF concentrations from those observed in plasma. Infection has an important influence on cytokine production, particularly in the case of IL-6. The data suggest that account should be taken of infection in patients where inflammatory markers are being evaluated and that IL-6 may be a valuable early indicator of CSF infection in patients with EVDs.

## Abbreviations

CSF: Cerebrospinal fluid; EVD: External ventricular drain; IL: Interleukin; Ra: Receptor antagonist; SAH: Subarachnoid haemorrhage; TNF: Tumour necrosis factor; VRI: Ventriculostomy-related infection.

## Competing interests

The authors declare that they have no competing interests.

## Authors’ contributions

SJH, AV, NJR and PT conceived the study and designed the protocols with CJM and ATK. Patients samples and details were collected by CJM, JG and SH. Samples were analysed by MH and SS, and data was analysed and interpreted by SJH, CJM, NS, MH, SS, HP, AV,SH and ATK. The manuscript was drafted by SJH, with input from all other authors, who also approved the final manuscript.
